# Quaternary Structure of Pathological Prion Protein as a Determining Factor of Strain-Specific Prion Replication Dynamics

**DOI:** 10.1371/journal.ppat.1003702

**Published:** 2013-10-10

**Authors:** Florent Laferrière, Philippe Tixador, Mohammed Moudjou, Jérôme Chapuis, Pierre Sibille, Laetitia Herzog, Fabienne Reine, Emilie Jaumain, Hubert Laude, Human Rezaei, Vincent Béringue

**Affiliations:** INRA (Institut National de la Recherche Agronomique), UR892, Virologie Immunologie Moléculaires, Jouy-en-Josas, France; Istituto Superiore di Sanità, Italy

## Abstract

Prions are proteinaceous infectious agents responsible for fatal neurodegenerative diseases in animals and humans. They are essentially composed of PrP^Sc^, an aggregated, misfolded conformer of the ubiquitously expressed host-encoded prion protein (PrP^C^). Stable variations in PrP^Sc^ conformation are assumed to encode the phenotypically tangible prion strains diversity. However the direct contribution of PrP^Sc^ quaternary structure to the strain biological information remains mostly unknown. Applying a sedimentation velocity fractionation technique to a panel of ovine prion strains, classified as *fast* and *slow* according to their incubation time in ovine PrP transgenic mice, has previously led to the observation that the relationship between prion infectivity and PrP^Sc^ quaternary structure was not univocal. For the *fast* strains specifically, infectivity sedimented slowly and segregated from the bulk of proteinase-K resistant PrP^Sc^. To carefully separate the respective contributions of size and density to this hydrodynamic behavior, we performed sedimentation at the equilibrium and varied the solubilization conditions. The density profile of prion infectivity and proteinase-K resistant PrP^Sc^ tended to overlap whatever the strain, *fast* or *slow*, leaving only size as the main responsible factor for the specific velocity properties of the *fast* strain most infectious component. We further show that this velocity-isolable population of discrete assemblies perfectly resists limited proteolysis and that its templating activity, as assessed by protein misfolding cyclic amplification outcompetes by several orders of magnitude that of the bulk of larger size PrP^Sc^ aggregates. Together, the tight correlation between small size, conversion efficiency and duration of disease establishes PrP^Sc^ quaternary structure as a determining factor of prion replication dynamics. For certain strains, a subset of PrP assemblies appears to be the best template for prion replication. This has important implications for fundamental studies on prions.

## Author Summary

Prions are infectious agents causing irremediably fatal neurodegenerative diseases in human and in farmed or wild animals. They are thought to be formed from abnormally folded assemblies (PrP^Sc^) of the host-encoded prion protein (PrP^C^). Different PrP^Sc^ conformational variants associated with distinct biological phenotypes, or ‘strains,’ can propagate in the same host. To gain some structural information on the physical relationship between packing order (i.e. quaternary structure) and the strain-specific biological information, we previously subjected PrP^Sc^ assemblies from prion strains classified as *fast* or *slow* (according to their survival time in susceptible laboratory animals) to sedimentation velocity ultracentrifugation experiments. For the *fast* strains specifically, the most infectious assemblies sedimented slowly and partitioned from the bulk of PrP^Sc^ macromolecular complexes. By changing the solubilization and sedimentation conditions, we established here that a small PrP^Sc^ aggregation size and not a low density accounts for these hydrodynamic properties. We further showed that these small assemblies resist proteolytic digestion and outcompete by several orders of magnitude the larger-size assemblies in cell-free prion conversion assays. Thus PrP^Sc^ quaternary structure appears to be a determining factor of prion replication dynamics. For certain strains, a discrete subset of PrP^Sc^ assemblies appears to be the best template for prion replication.

## Introduction

Prion disease pathogenesis stems from the post-translational conversion of the monomeric, alpha helix-rich host-encoded prion protein (PrP^C^) into misfolded, β sheet-enriched PrP^Sc^ aggregates [1]. The process is believed to be initiated by PrP^Sc^ seeds [2], [3] acquired through infection or arising from spontaneous conversion of wild-type or mutant PrP^C^ into PrP^Sc^ [4]. The PrP^Sc^ seeds would template the remodeling of host PrP^C^ to the PrP^Sc^ form [5]. This self-sustained polymerization process, -in which polymer fragmentation is thought to play a key role [2], [6], [7]-, leads to deposition of injurious plaques into the brain. PrP^Sc^-templated conversion of PrP^C^ or bacterially-derived PrP has been established in cell-free conditions using protein misfolding cyclic amplification (PMCA) assays (for reviews [8], [9]), further strengthening the conformational changes of the prion protein as the main molecular determinant of prion replication and infectivity.

Prion diseases can occur in many mammalian species. Among them are human with Creutzfeldt-Jakob disease, sheep and goat with scrapie, cattle with bovine spongiform encephalopathy (BSE) and cervids with chronic wasting disease [10]. A variety of prion variants or strains exist within a given host species. They cause diseases with specific phenotypic traits, including time course to disease and neuropathological features. Differences in PrP^Sc^ biochemical (e.g. resistance to proteases) and biophysical properties [11], [12], [13], [14], [15] indicate that strain-specific biological properties reflect differences in the PrP^Sc^ “conformation” associated to each strain [16], [17], [18]. PrP^Sc^ has not been amenable to high-resolution structural studies [3], due notably to its insolubility in non-denaturing detergents. Thus the conformational underpinnings of the prion strain phenomenon and notably the contribution of PrP^Sc^ quaternary structure remain largely elusive. Conceivably these differences must be sufficiently local to allow faithful prion transmission at least within and between individuals of the same species. Non-PrP components might be part of prion infectious particle or act as a scaffold during the conversion and/or aggregation process and thus might also contribute to prion strain biological phenotype (reviews: [3], [19]).

To gain some structural information on the physical relationship between prion infectivity and PrP^Sc^ aggregation state, and how it varies among strains, we previously applied a sedimentation velocity (SV)-based fractionation technique to solubilized brain homogenates from ovine PrP *tg338* transgenic mice infected with distinct scrapie and BSE cloned prion strains [20]. Based on the incubation time to disease in *tg338* animals, these strains were classified as *fast* and *slow*. These experiments led to the observation that the relationship between prion infectivity and PrP^Sc^ aggregation state was not univocal. Regardless of the strain, the bulk of proteinase-K (PK) resistant PrP^Sc^ was found to sediment in the middle part of the gradient. While for the *slow* strains, the distribution of infectivity tended to correlate with that of PK-resistant PrP^Sc^, for the *fast* strains specifically, infectivity peaked markedly in the upper top gradient fractions, which were much less populous in PK-resistant PrP^Sc^ aggregates. Although SV is known to separate protein aggregates according essentially to their size, density can often influence their sedimentation properties, thus questioning which parameters would account for the hydrodynamic properties of the *fast* strain most infectious component.

Here, fractioning the same ovine strains by sedimentation equilibrium (SE) demonstrates that the density properties of prion infectivity and PK-resistant PrP^Sc^ tend to overlap regardless of the strain, *fast* or *slow*, and the solubilization conditions. This indicates that a reduced PrP^Sc^ aggregation size and not a low density essentially account for the SV properties of the most infectious assemblies from the *fast* strains. We further show that these SV-isolable, small sized infectious assemblies perfectly resist limited protease-induced proteolysis and that their templating activity by PMCA outcompetes that of the bulk of larger size aggregates by several orders of magnitude.

## Material and Methods

### Ethics statement

Animal experiments were carried out in strict accordance with EU directive 2010/63 and were approved by the authors' institution local ethics committee (Comethea; permit number 12/034).

### Prion strains

The cloned ovine prion strains used in this study have been previously described [20]. They have been obtained through serial transmission and subsequent biological cloning by limiting dilutions of classical and atypical field scrapie and experimental sheep BSE sources to *tg338* transgenic mice expressing the VRQ allele of ovine PrP. Pooled or individual *tg338* mouse brain homogenates (20% wt/vol. in 5% glucose) were used in centrifugation analyses, as indicated.

### Velocity and equilibrium sedimentation

The entire, standard procedure was performed at 4°C unless specified otherwise. Mouse brain homogenates were solubilized by adding an equal volume of solubilization buffer (50 mM HEPES pH 7.4, 300 mM NaCl, 10 mM EDTA, 2 mM DTT, 4% (wt/vol.) dodecyl-β-D-maltoside (Sigma)) and incubated for 45 min on ice. Sarkosyl (N-lauryl sarcosine; Fluka) was added to a final concentration of 2% (wt/vol.) and the incubation continued for a further 30 min on ice. For SV, a volume of 150 µl was loaded on a 4.8 ml continuous 10–25% iodixanol gradient (Optiprep, Axys-shield), with a final concentration of 25 mM HEPES pH 7.4, 150 mM NaCl, 2 mM EDTA, 1 mM DTT, 0.5% Sarkosyl. For SE, a volume of 220 µl was mixed to reach 40% iodixanol, 25 mM HEPES pH 7.4, 150 mM NaCl, 2 mM EDTA, 1 mM DTT, 0.5% Sarkosyl final concentration and loaded within a 4.8 ml of 10–60% discontinuous iodixanol gradient with a final concentration of 25 mM HEPES pH 7.4, 150 mM NaCl, 2 mM EDTA, 1 mM DTT, 0.5% Sarkosyl.

The gradients were centrifuged at 285 000 g for 45 min (SV) or at 115 000 g for 17 hours (SE) in a swinging-bucket SW-55 rotor using an Optima LE-80K ultracentrifuge (Beckman Coulter). We found that 5 hours was the minimum time to run proteins at the equilibrium in the optiprep medium. Gradients were then manually segregated into 30 equal fractions of 165 µl from the bottom using a peristaltic pump. Fractions were aliquoted for immunoblot, bioassay or scrapie cell assay analyses. Gradient linearity was verified by refractometry. To avoid any cross-contamination, each piece of equipment was thoroughly decontaminated with 5 M NaOH followed by several rinses in deionised water after each gradient collection. To ascertain the efficiency of the decontamination procedure, solubilized, uninfected brain homogenates were fractionated at the equilibrium. Some resulting fractions were inoculated to *tg338* mice (see below). Those were euthanized healthy at 500 days post-inoculation. Their brain and spleen were negative for PrP^Sc^ content.

### Solubilization with digitonin, saponin and cylodextrin

Digitonin (0.1% final concentration; Sigma) or saponin (0.5% final; Sigma) or methyl-β cyclodextrin (10 mM final; Sigma) were added before or after solubilization with dodecyl-β-D-maltoside and Sarkosyl. The incubation was performed for further 30 min on ice.

### Proteinase K digestion before sedimentation velocity fractionation

Brain homogenates from *tg338* mice infected with LA21K *fast* prions (20% wt/vol. in 5% glucose) were adjusted to a final concentration of 25, 50 and 100 µg/ml proteinase K and incubated under constant agitation at 37°C for 1 hour. The digestion was blocked with phenylmethylsulfonyl fluoride (10 mM final concentration; Roche). Undigested samples treated in the same conditions were used as controls. The samples were solubilized and fractionated by SV as described above. The fractions were then inoculated to *tg338* reporter mice to estimate their infectivity (see below).

### Analysis of PrP^C^, PrP^Sc^, PK-resistant PrP^Sc^ and other protein content by immunoblot

Aliquots of the collected fractions were treated or not with a final concentration of 50 µg/ml PK (1 hour, 37°C). Samples were then mixed in Laemmli buffer and denatured at 100°C for 5 min. The samples (15 µl) were run on 12% Bis-Tris Criterion gels (Bio-Rad, Marne la Vallée, France) and electrotransferred onto nitrocellulose membranes. In some instances, denatured samples (100 µl) were spotted onto nitrocellulose membranes using a dot-blot apparatus (Schleicher & Schuell BioScience (Whatman)). Nitrocellulose membranes were probed for PrP with 0.1 µg/ml biotinylated anti-PrP monoclonal antibody Sha31 as previously described [20]. Thy.1, flotillin and caveolin proteins were probed with anti-CD90.1 (Southern Biotec), anti-flotillin-1 (Abcam) and anti-caveolin-1 (Abcam) antibodies, respectively. Immunoreactivity was visualized by chemiluminescence (GE Healthcare). The amount of PrP present in each fraction was determined by the GeneTools software after acquisition of chemiluminescent signals with a GeneGnome digital imager (Syngene, Frederick, Maryland, United States). The PrP sedimentation profiles obtained by immunoblot were normalized to units and decomposed using multiple Gaussians fits procedures with a maximum entropy minimization approach.

### Mouse bioassay for infectivity titration

Fractions (unless specified otherwise) were diluted extemporarily in 5% glucose (1∶5) in a class II microbiological cabinet according to a strict protocol to avoid any cross-contamination. Individually identified 6- to 10-week old *tg338* recipient mice (n≥5 mice per fraction) were inoculated intracerebrally with 20 µl of the solution, using a 26-gauge disposable syringe needle inserted into the right parietal lobe. Mice showing prion-specific neurological signs were monitored daily and euthanized at terminal stage of disease. To confirm prion disease, brains were removed and analyzed for PK-resistant PrP^Sc^ content using the Bio-Rad TsSeE detection kit [21] before immunoblotting, as above. The survival time was defined as the number of days from inoculation to euthanasia. To estimate what the difference in mean survival times means in terms of infectivity, strain-specific curves correlating the relative infectious dose to survival times were used, as previously described [20].

### Rov-scrapie cell assay for infectivity titration

The Rov-cell assay technique will be published elsewhere. Gradient fractions aliquots (20–30 µl) were methanol precipitated as done previously [20], before resuspension in Rov cells culture medium. We verified that methanol precipitation did not affect the overall infectious titer of the samples to titrate. Rov cell [22] monolayers established in a 96 well plate were exposed to the fractions for one week. After several washes with sterile PBS, the cells were further cultivated for two weeks before fixation and PrP^Sc^ detection by immunofluorescence using the ICSM33 anti-PrP antibody (D-Gen Ltd, [23]). Immunofluorescent PrP^Sc^ signals were acquired with an inverted fluorescence microscope (Zeiss Axiovision). The signal was quantified per cell per well, as previously described [20]. Serial tenfold dilutions of infected brain homogenates were prepared in the same conditions and run in parallel experiments to establish a tissue culture infectious dose curve that directly relates to the PrP^Sc^ content.

### PMCA

The modified PMCA procedure will be published elsewhere. It has been adapted from previously described protocols [24], [25]. The PMCA substrate was composed of 10% (wt/vol.) *tg338* brain homogenate in PMCA buffer (Tris-HC 50 mM pH 7.4, 1% Triton X-100, 150 mM NaCl). Serial ten-fold dilutions of fractions either as pool or individuals were mixed with substrate lysate in 0.2 ml thin-wall PCR tubes containing beads. Tubes were placed in the Misonix S3000 or Q700 sonicator horns (Misonix, Farmingdale USA; Delta Labo, France) for a round of 96 cycles. Each cycle consisted of a 30 s sonication step at ∼200–250 W followed by a 29.5 min incubation at 37°C. Negative controls were run in parallel. They were composed of unseeded substrate or seeded with uninfected fractions. Aliquots of the amplified samples were digested with PK (100 µg/ml final concentration) for 1 h at 37°C before denaturation in Laemmli sample buffer and dot- or western-blot analysis as described above.

## Results

### LA21K *fast* prions conserve distinct infectivity and PrP^Sc^ SV profiles in more stringent solubilization and ultracentrifugation conditions

**Figure 1:**
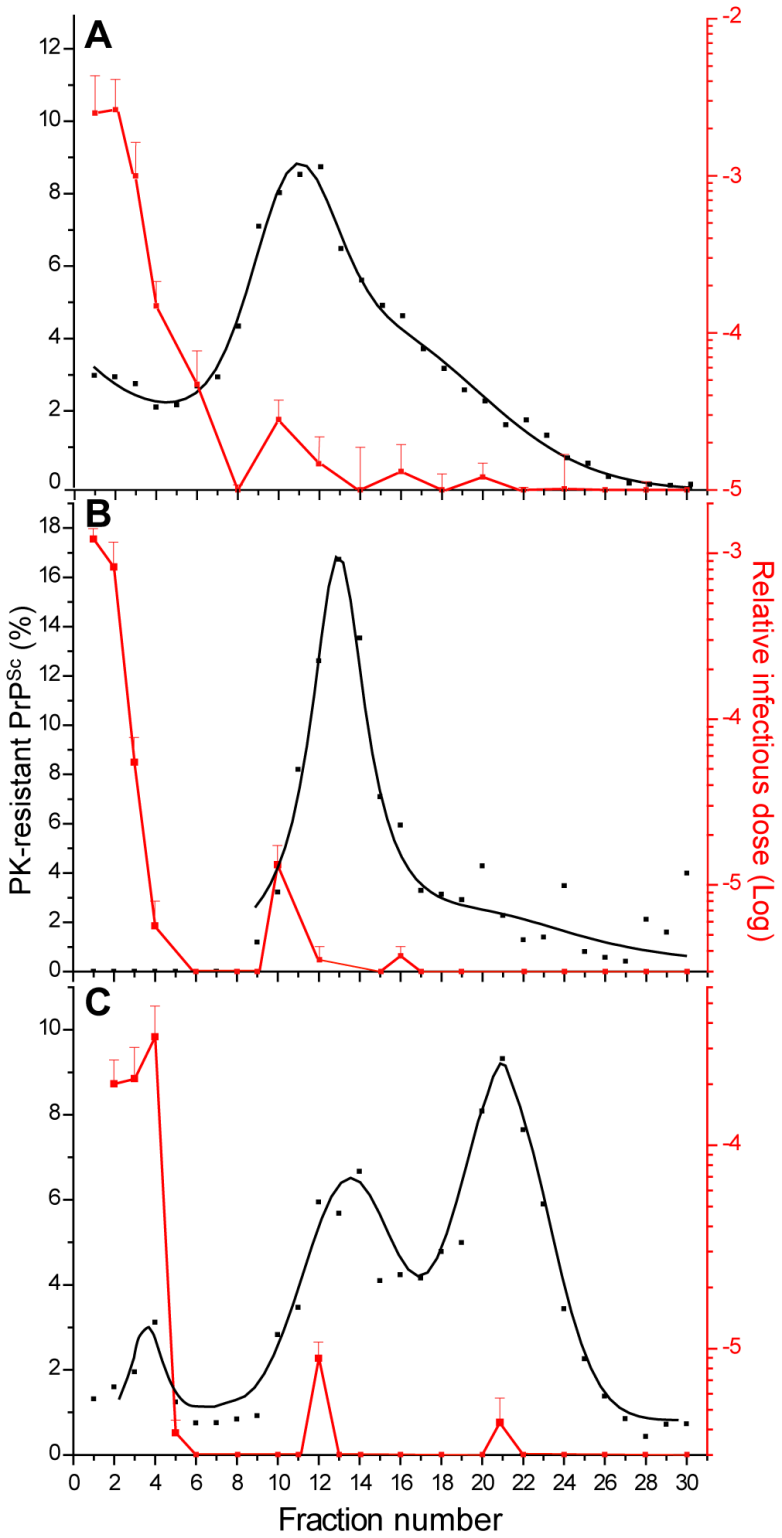
Effect of solubilization temperature and increase in ultracentrifugation time on the sedimentation velocity profile of PK-resistant PrP^Sc^ and infectivity. Brain homogenates from *tg338* mice infected with LA21K *fast* prions were solubilized at 4°C (**A**; **C**) or 37°C (**B**), loaded on top of a linear optiprep gradient and ultracentrifuged for 45 min (**A**, **B**) or 90 min (**C**). Fractions collected from the gradient (numbered from top to bottom) were analyzed for PK-resistant PrP^Sc^ content (black line; left axis) and for infectivity (red line; right axis). The mean levels of PK-resistant PrP^Sc^ per fraction have been obtained from the immunoblot analysis of n≥3 independent fractionations. As the replicates gave consistent results, these data were combined and fit. Fraction infectivity was determined by a Rov cell assay. It is based on the quantification of PrP^Sc^-containing Rov cells by immunofluorescence using PrP^Sc^-specific antibodies. The cells were exposed in parallel to fraction aliquots and to serial tenfold dilutions of a LA21K *fast*-infected brain (expressed as relative infectious doses) prepared in the same conditions. The data presented are the mean ± SEM of n = 3 independent titrations. Data presented in A are from ref. [20] and this study.

PrP^Sc^ and infectivity from *fast* prion strains exhibited dissimilar hydrodynamic properties by SV, the most infectious assemblies sedimenting slowly [20]. While the detergent used to solubilize brain homogenates disrupted the membrane integrity and released PrP^C^ in the soluble phase [20], -suggesting efficient solubilization conditions-, a tight and specific association of *fast* prion strains infectivity with lipids, which would also float in the gradient upon ultracentrifugation, could not be totally excluded. To address this possibility, we examined the distribution of LA21K *fast* infectivity in more stringent solubilization conditions, with the detergents dodecyl maltoside and sarkosyl used sequentially at 37°C instead of 4°C [26], before standard SV fractionation in an iodixanol (Optiprep) gradient [20]. For each fraction, PK-resistant PrP^Sc^ was detected by immunoblot and infectivity was measured with a Rov cell-based assay, as previously described [20]. As a result, solubilization at 37°C did not significantly modify the distribution of infectivity in the gradient: the most infectious fractions were found in the top of the gradient, fractions 1 and 2 being 100–1000 fold more infectious than the middle fractions 12–16 containing the bulk of PK-resistant PrP^Sc^ (**Figures 1 A–B**).

To gain resolution in the SV profile, the ultracentrifugation time was doubled. As shown in **Figure 1 C**, the infectivity peak shifted from fraction 1–2 to fractions 2–4 while PK-resistant PrP^Sc^ was found to sediment toward the heaviest fractions of the gradient [12]–[26]. However the shift of infectivity downward was considered as too slight to firmly exclude an intrinsically low density. We therefore decided to study the density of PrP^Sc^ and infectivity of the *fast* strains by sedimentation at the equilibrium. This was compared to that of the *slow* strains, for which infectivity and PK-resistant PrP^Sc^ SV profiles overlapped [20]. Sedimentation equilibrium (SE) allows macromolecules reaching a position in the centrifuge tube at which their own density equals that of the gradient density, independent of time. To achieve this, the sample is mixed with the gradient material (encompassing a wider range of densities than for SV) and the sample is run for a long period of time (reviewed in [27]).

### ‘Fast’ and ‘slow’ prion strains exhibit overlapping PrP^Sc^ and infectivity density distribution profiles

**Figure 2:**
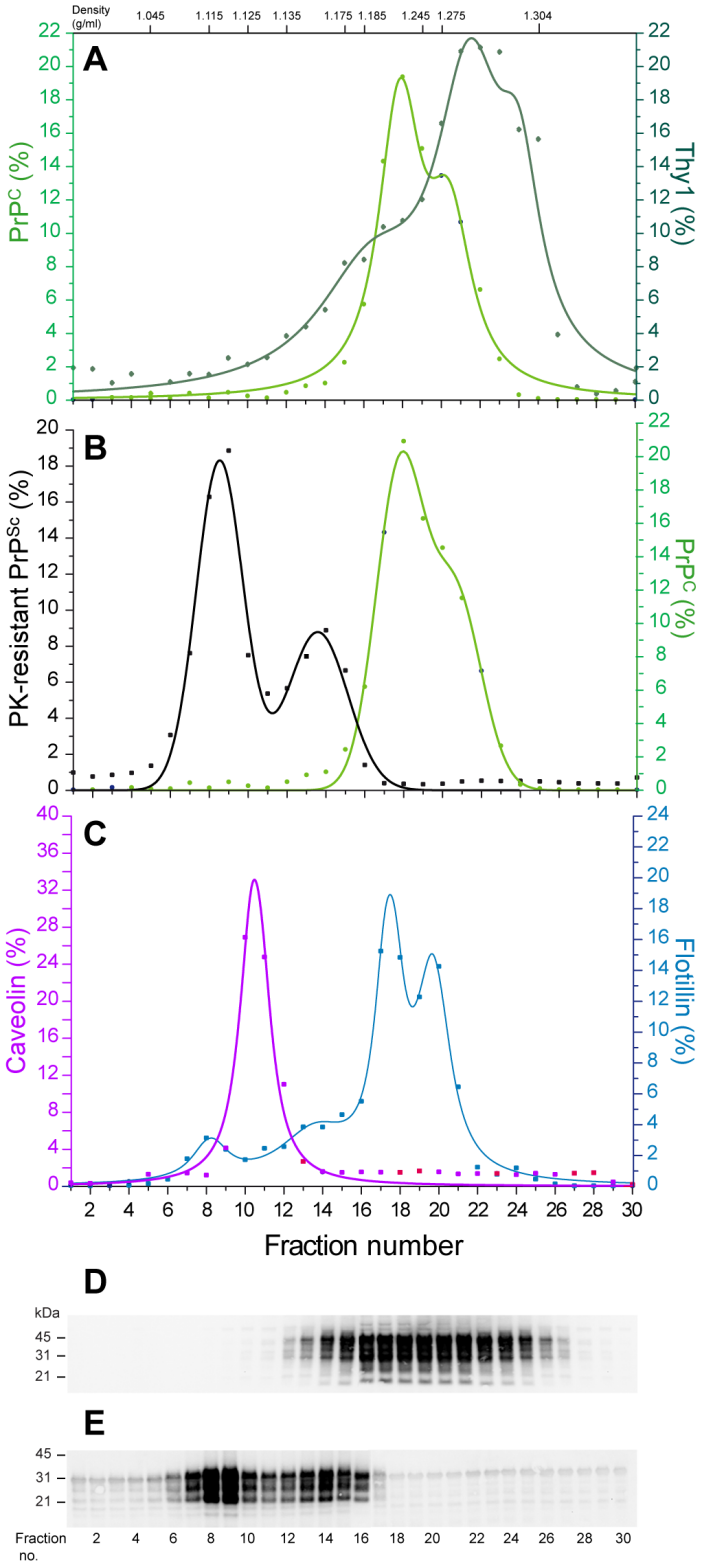
Immunoblot analysis of PrP material from *tg338* mouse brain sedimented at the equilibrium. Solubilized brain material from uninfected (**A, D**) or 127S (**B**, **C, E**) infected *tg338* mice was fractionated by sedimentation at the equilibrium. The collected fractions (numbered from top to bottom of the gradient) were analyzed for PrP^C^ (**A**; **B**), Thy1 (**A**), flotillin and caveolin (**C**) content by immunoblot without (**A**; **B**, right axis; **C**) or after PK digestion (**B**, left axis). The relative amounts of those proteins per fraction were reported on the graphs, as indicated on left or right axis. The data presented are the mean ± SEM of n≥3 independent fractionations. The density values estimated by refractometry are indicated on the top of the graph. (**D**, **E**) Representative immunoblots of PrP^C^ (**D**) and PK-resistant PrP^Sc^ (**E**) distribution at the equilibrium are shown.

To separate PrP assemblies by density, solubilized brain homogenates were centrifuged isopynically in 10–60% discontinuous iodixanol gradient for 17 hours at 115 000 g. The gradient was then fractionated in 30 fractions of equivalent volume and PrP distribution was assessed by immunoblotting. Three or more independent fractionation experiments with different pooled or individual brains were performed for each strain to assess the reproducibility of the partition and to enable quantitative analysis of the data. In uninfected (**Figure 2A, D**) as in infected brain (**Figure 2B, E**) homogenates, PrP^C^ was found in fractions 14–26 and peaked in fraction 18–20, i.e. at a density of ∼1.23–1.28 g/ml (**Figure 2A**). Other GPI and/or lipid rafts-associated proteins such as Thy1 and flotillin were found in the PrP^C^-enriched fractions or in the vicinity (**Figure 2 A,C**), further supporting the view that the conditions employed here led to efficient solubilization of proteins present in detergent resistant microdomains.

**Figure 3:**
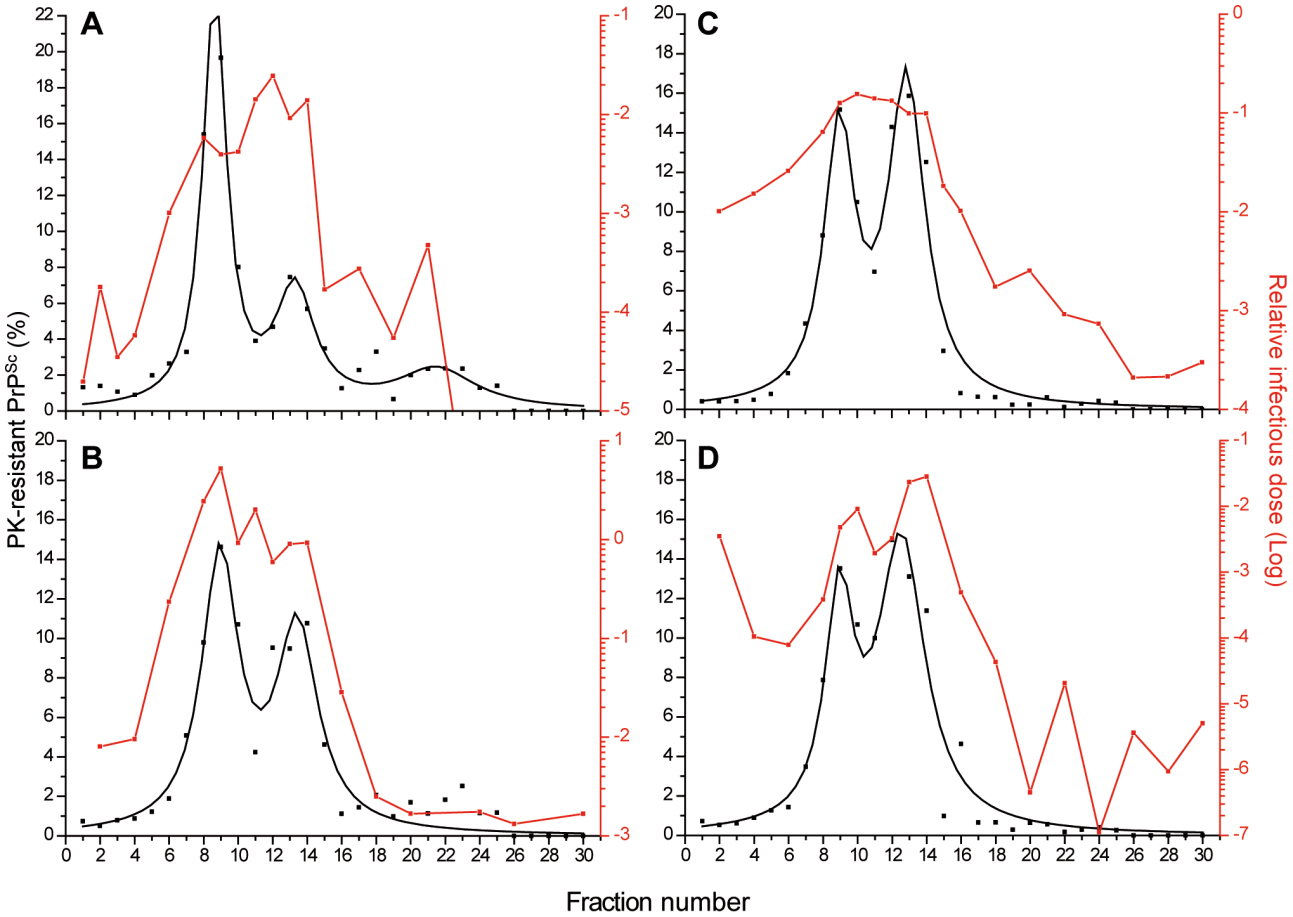
Overlapping PK-resistant PrP^Sc^ and infectivity density profiles of prion strains. Brain homogenates from *tg338* mice infected with LA21K *fast* (**A**), LA19K (**B**), sheep BSE (**C**) and Nor98 (**D**) were solubilized and fractionated by sedimentation at the equilibrium. The fractions collected from the gradient were analyzed for PK-resistant PrP^Sc^ content (black line) and for infectivity (red line). The mean levels of PK-resistant PrP^Sc^ per fraction shown are the combined and fit replicates (left axis) obtained from the immunoblot analysis of n≥3 independent fractionations. For each fraction analyzed, infectivity was determined by applying the mean survival times values in *tg338* mice (Table 1) to standard dose response curves established for each strain [20]. The right, logarithmic scale provides the reciprocal relative infectious dose found. A relative infectious dose of 0 corresponds to animal inoculated with the equivalent of 2 mg of infectious brain tissue.

The combined curves resulting from the replicate analysis of PrP content indicated that PK-resistant PrP^Sc^ aggregates from five ovine strains, - two *fast* strains, 127S (**Figure 2B**) and LA21K *fast* (**Figure 3A**) and 3 *slow* strains, LA19K, sheep BSE and Nor98 (**Figure 3 B–D**) -distributed in two major populations peaking in fractions 8–10 and 12–14, i.e. at respective density of ∼1.115 and ∼1.145 g/ml, nearby that of caveolin, another lipid rafts resident, but oligomeric protein (**Figure 2C**). Only the proportion of PK-resistant PrP^Sc^ per peak varied to a significant degree among the strains.

The distribution of infectivity was assessed by a *tg338* mouse incubation time bioassay, using one fractionation performed with pooled brains. It was repeated partially with one strain (Nor98) to confirm the reproducibility of the method. In striking contrast with SV [20], the distribution of infectivity at the equilibrium broadly overlapped that of PK-resistant PrP^Sc^, whether the strain was *fast* or *slow*. Thus, for all the strains, fractions 8 to 14 were the most infectious, based on the mean survival times of the mice that succumbed to disease (**Table 1**). The mean survival times of mice inoculated with the fractions at the two PrP^Sc^ density peaks rarely differed to a significant level (**Figure S1**). Standard infectious dose/survival time curves established individually for each strain tested here [20] indicated that the fractions of higher density were at least 100–1000 less-fold infectious than the most infectious fractions (**Figure 3**). There was some strain-dependent variation in the distribution of infectivity in the top fractions of very low density (**Figure 3**). While for LA21K *fast*, LA19K and sheep BSE the differences in survival times between the upper top fractions 1–4 and the most infectious fractions 8–14 were statistically significant, those did not always reach significant values for Nor98 (**Figure S1**). For the LA21K *fast* strain, this provided a 100 to 1000-fold difference in infectious titer between the top and most infectious fractions (**Figure 3A**). For this strain, the cumulated infectivity of the most infectious fractions by SE approached that previously found in the top fractions by SV [20]. This further supported the view that the most infectious population isolated by SV was indeed present in the middle of the SE gradients.

**Table 1:** Mean survival time of *tg338* mice intracerebrally inoculated with prion strains fractionated by sedimentation at the equilibrium.

	LA21K *fast*		127S		Nor98		Nor98		LA19K		BSE	
Fractions	n/n_0_ 1	Survival^2^	n/n_0_ 1	Survival^2^	n/n_0_ 1	Survival^2^	n/n_0_ 1	Survival^2^	n/n_0_ 1	Survival^2^	n/n_0_ 1	Survival^2^
**1**	5/5	98±4										
**2**	4/4	87±2	5/5	93±2	6/6	283±9	5/5	273±5	6/6	210±15	6/6	183±2
**3**	5/5	95±3	5/5	86±2								
**4**	5/5	92±1	5/5	85±1	6/6	287±13	6/6	287±8	6/6	206±11	6/6	177±1
**6**	5/5	80±1			6/6	268±13			5/5	163±11	6/6	169±1
**8**	5/5	75±1	5/5	78±2	6/6	242±8			6/6	146±3	6/6	158±2
**9**	5/5	76±1	4/4	75±1	5/5	245±4	6/6	264±9	6/6	142±1	6/6	151±2
**10**	5/5	75±1	5/5	74±1	6/6	236±4	5/5	246±6	6/6	152±2	6/6	149±2
**11**	4/4	72±1			6/6	251±2			6/6	147±9	6/6	150±2
**12**	5/5	70±2	5/5	69±2	6/6	246±10	6/6	246±7	6/6	156±1	5/5	151±1
**13**	5/5	73±1			6/6	227±10			6/6	153±3	5/5	155±3
**14**	5/5	72±1			6/6	227±10	6/6	222±15	6/6	152±2	6/6	154±2
**15**	4/4/	87±3			6/6	226±14					9/9	174±3
**16**					6/6	266±17			6/6	186±6	5/5	183±4
**17**	5/5	85±2										
**18**					6/6	295±10			6/6	254±46	7/7	220±6
**19**	5/5	93±1										
**20**					6/6	320±14	4/4	303±12	4/4/	292±23	4/4	211±13
**21**	4/4	102±4										
**22**					6/6	303±14			1/6	329	5/6	243±19
**23**	3/5	130±18										
**24**					5/6	384±45	3/6	380±11	1/6	244	5/6	252±26
**25**	0/5	>400										
**26**					6/6	327±7			1/6	307	2/6	218; 497
**27**	0/5	>400										
**28**									0/6	>600	1/6	322
**29**	1/5	189										
**30**					6/6	322±6			1/6	182	4/6	271±21

1 n/n_0_: Number of diseased, brain PrP^res^-positive/inoculated mice.

2 Mean survival time of the infected mice in days ± SEM.

**Figure 4:**
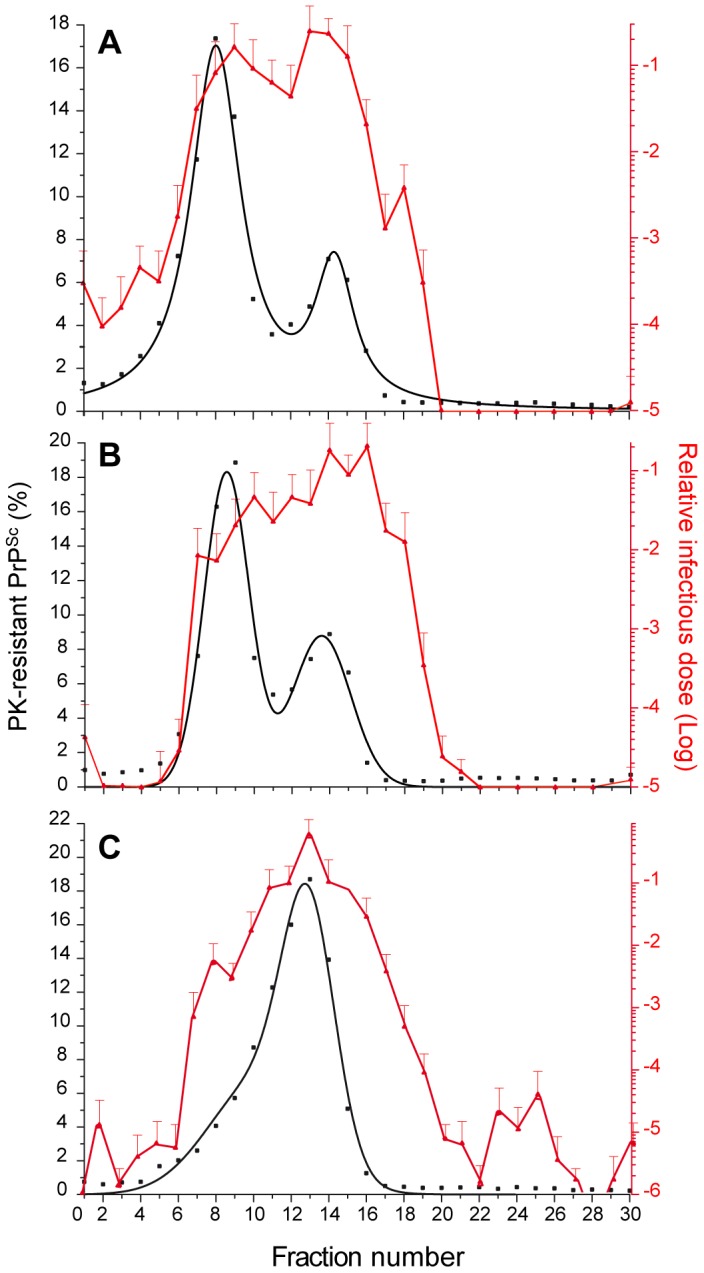
Overlapping PK-resistant PrP^Sc^ and infectivity density profiles of ‘fast’ prions upon additional solubilization with digitonin. Brain homogenates from *tg338* mice infected with LA21K *fast* (**A**) or 127S (**B**, **C**) were solubilized in the standard conditions (**A**, **B**) or by adding digitonin first (**C**) before fractionation by sedimentation at the equilibrium. The fractions collected from the gradient were analyzed for PK-resistant PrP^Sc^ content (black line; left axis) and for infectivity (red line; right axis). The mean levels of PK-resistant PrP^Sc^ per fraction shown are the combined and fit replicates obtained from the immunoblot analysis of n≥3 independent fractionations. Fraction infectivity was determined by a Rov cell assay as described in Figure 1 (mean ± SEM of n = 3 independent titrations).

The SE distribution profile of LA21K *fast* infectivity was similar when the mouse incubation time bioassay was substituted with the Rov cell assay (n = 3 independent experiments, compare **Figure 3A** and **Figure 4A**). Thus differences in survival times were correlated with differences in infectivity content and not different pathogenic effects. The infectivity distribution profile associated with the other *fast* strain, 127S was closely related to that of LA21K *fast* (**Figure 4B**), as measured by the scrapie cell assay (n = 3 independent fractionation studies; **Figure 4B**) or partly by the incubation time bioassay (**Table 1**). For both LA 21K *fast* and 127S, the relative infectious levels at the two PrP^Sc^ density peaks rarely differed one from the other significantly, as estimated by the Rov cell assay (**Figure S2**).

Collectively, these data showed a good correlation between the density profile of infectivity and that of PK-resistant PrP^Sc^ aggregates, regardless the “speediness” of the prion strain.

### The infectivity and PrP^Sc^ density distribution profiles of 127S prions evolve similarly upon additional solubilization with digitonin

To further ascertain that the relative overlap, at the equilibrium, in the distribution of PrP^Sc^ and infectivity of the *fast* strains truly reflects a physical association with respect to density, we studied the impact on their sedimentation profile of alterations in the solubilization procedure. We added saponin or digitonin (two closely related detergents) or the drug methyl-β cyclodextrin before or after the solubilization with dodecyl maltoside and sarkosyl. These agents are known to specifically deplete or sequester membrane lipids such as cholesterol [28], [29], [30]. The solubilization was performed at either 4°C or 37°C to increase the treatment stringency. This was tested on the 127S *fast* strain. None of the molecules tested modified PrP^C^ sedimentation profile (data not shown). Only digitonin modified the distribution profile of PK-resistant PrP^Sc^ at the equilibrium. The peak of lower density in fraction 8–10 was blurred leading to a Gaussian-like distribution of the protein centered in fraction 13 (**Figure 4C**). This digitonin effect was observed at 4°C and 37°C, independently of the order in which the detergent was used (data not shown). Adding digitonin to the solubilization procedure led to the evolution of 127S infectivity density profile towards a single peak consistently associated with PK-resistant PrP^Sc^ (n = 3 experiments, **Figure 4C**). Such effect was not observed with saponin and methyl-β cyclodextrin (data not shown). Together these data further reinforces the view that the density of PrP^Sc^ and infectivity of the *fast* prions strains are physically associated.

To conclude with SE experiments, all the data gained using this technique concur to the view that small size and not low density is mostly responsible for the distinctive hydrodynamic properties of the *fast* strain most infectious component by SV and its partitioning from the bulk of PrP^Sc^.

### PK resistance of the infectivity associated with, SV-fractionated PrP^Sc^ aggregates from LA21K ‘fast’ prions

Having undoubtedly identified that PrP^Sc^ aggregates from the *fast* strains segregated in two populations of differing size and infectivity level by SV, we next examined their respective resistance to PK treatment. This was motivated by the low content of PK-resistant PrP^Sc^ of the most infectious population (<10%; **Figure 1** and [20]) and the reported existence of small sized PK-sensitive aggregates [31], [32]. LA21K *fast* brain homogenates were treated with concentrations of PK (0–100 µg/ml) for 1 hour at 37°C prior to SV fractionation. These concentrations were chosen to completely digest PrP^C^ while preserving PK-resistant PrP^Sc^ ([21] and unpublished observations). The most infectious fractions (1+2) and the fractions in which PK-resistant PrP^Sc^ levels were peaking (12+13) were then pooled, respectively, and inoculated to reporter *tg338* mice to assess their relative infectivity levels by incubation time bioassay. This was done in two independent experiments summarized in **Table 2**. In both experiments, the mean survival time of mice inoculated with the top fractions was marginally prolonged upon the different PK treatments. It would correspond to a reduction <0.5 Log_10_ of the infectious titer. In contrast, the mean survival time of mice inoculated with the middle fractions was increased by 7 to 18 days upon PK treatment, i.e. a potential reduction of infectivity of >1 Log_10_. Together these data did not reveal an unusual susceptibility to PK of the LA21K *fast*, small size most infectious assemblies. The effect of PK treatment appeared even more significant on the larger size PrP^Sc^ assemblies.

**Table 2:** Survival time of *tg338* mice intracerebrally inoculated with LA21K *fast* prions treated with a range of PK concentrations before fractionation by sedimentation velocity.

Fractions inoculated		Experiment 1		Experiment 2	
	PK (µg/ml)	n/n_0_	Survival time (days ± SEM)	n/n_0_	Survival time (days ± SEM)
**1+2**	0	7/7	78±3.5	6/6	79±3.0
	25	6/6	83±3.5	nd	
	50	6/6	81±1.0	6/6	81±1.2
	100	6/6	77±3.0	5/5	82±3
**12+13**	0	5/5	89±1.0	5/5	90±2
	25	5/5	101±3	nd	
	50	4/5	105±5	5/5	97±3
	100	5/5	107±2	5/5	99±3

n/n_0_: Number of diseased, brain PrP^res^-positive/inoculated mice.

nd: not done.

### Templating efficiency of SV-fractionated PrP^Sc^ assemblies from LA21K ‘fast’ prions

**Figure 5:**
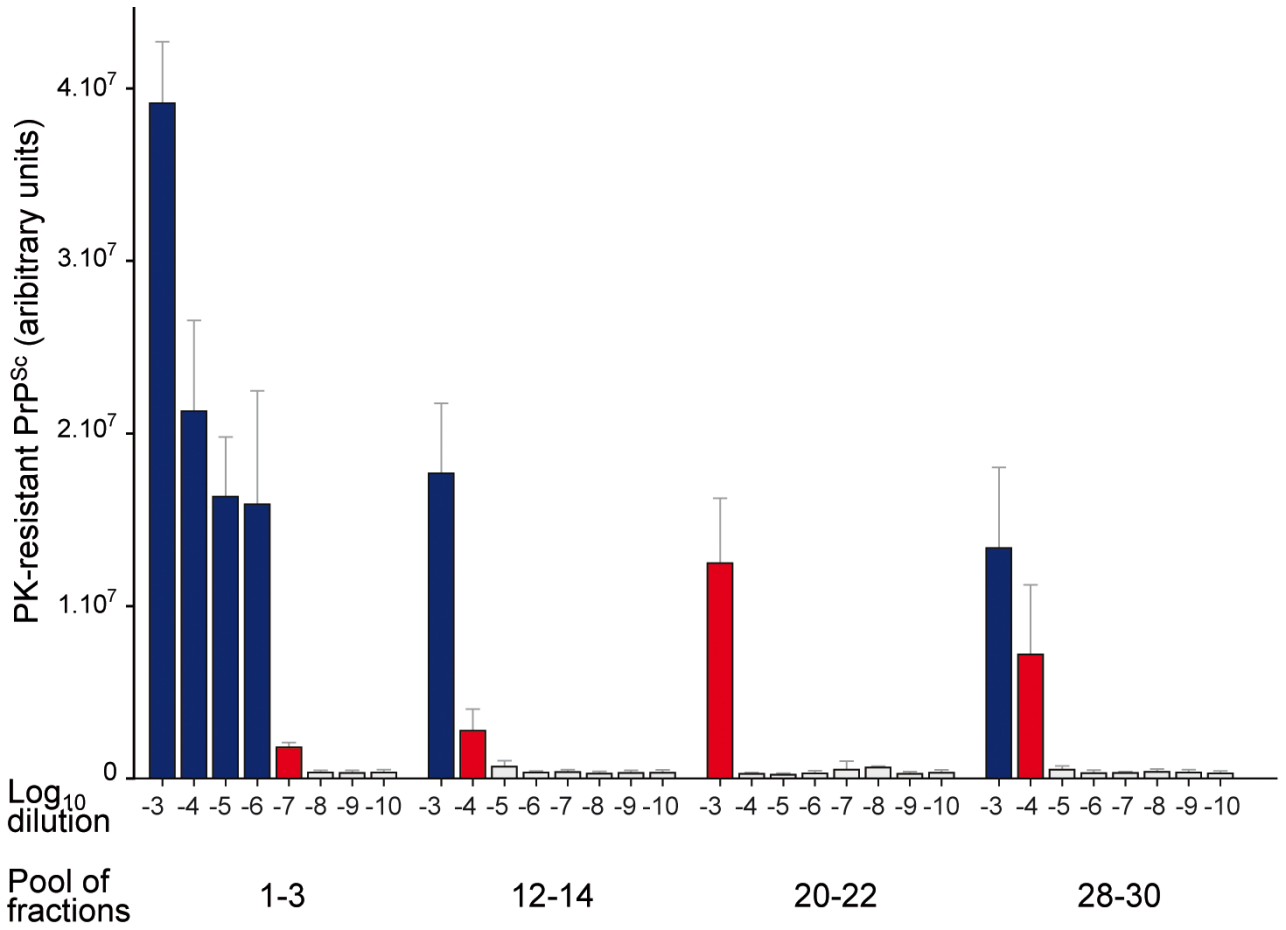
Templating activity of sedimentation velocity fractions of ‘fast’ prions. Brain homogenates from *tg338* mice infected with LA21K *fast* prions were solubilized in the standard conditions before fractionation by sedimentation velocity. The fractions collected from the gradient were pooled as indicated and serially diluted 10^3^ to 10^10^-fold before being used as templates in PMCA reaction (one round). The resulting product was PK-digested, denatured and quantified by immunoblot analysis. The data shown are the mean ± SEM levels of PK-resistant PrP^Sc^ from n = 4 independent experiments. The last positive dilution is indicated in red. The white bars are considered as nonspecific background.

SV fractionation and the PMCA technique were used to compare the templating efficiency of LA21K *fast* PrP^Sc^ assemblies with differing size and infectivity levels. Serial ten-fold dilutions of the upper most infectious fractions [1]–[3], intermediate PK-resistant PrP^Sc^ enriched fractions [12]–[14] and heavy [20]–[22], [28]–[30] fractions were mixed with uninfected *tg338* brain lysate and run for one PMCA round of 48 hours. Four independent experiments were performed using four independent fractionations. In each experiment, fractions were amplified in triplicates. The PMCA products were then treated with PK and analyzed by dot-blot based immunoblotting (**Figure 5**). A positive PrP^res^ signal was observed after PMCA amplification of the upper fractions 1–3 diluted up to 10^6^–10^7^-fold. In sharp contrast, no PrP^res^ signal was detected when the other pools of fractions were diluted more than 10^4^-fold before the PMCA reaction. Assuming a straight correlation between PMCA activity of the fractions and PrP assemblies' content, the specific templating activity per unit PrP^res^ would be 1000 to 10 000-fold higher for the discrete population of ‘small’ PrP^Sc^ oligomers than for the bulk of higher size PrP^Sc^ assemblies.

## Discussion

Our initial SV studies revealed striking divergence in the hydrodynamic properties of the most infectious assemblies between distinct ovine prion strains from the same host species. For *fast* strains specifically, the most infectious assemblies sedimented slightly and were associated with low levels of PK-resistant material ([20] and this study). To carefully separate the respective contributions of size and density to these hydrodynamic characteristics, we varied the solubilization conditions and performed sedimentation at the equilibrium. Incidentally this is the first study that compared the density of prion particles associated with phenotypically distinct strains propagated on the same genetic background. All these experiments concurred with the view that a reduced aggregation size but not a low density accounts for the low SV properties of the *fast* strain most infectious component. We also provided evidence that these SV-isolated, small sized infectious species resist limited PK-proteolysis and have high templating efficiency as suggested by PMCA assay. Together, the straight relationship between small sized PrP assemblies, conversion efficacy and short incubation time observed for the *fast* strains establishes PrP^Sc^ quaternary structure as a determining factor of prion (strain specific) replication dynamics.

Running the ovine prion strains at the equilibrium revealed that PrP^Sc^ sedimented in two major density peaks, their respective proportions varying among *fast* and *slow* strains. The density values of the 2 PrP^Sc^ peaks were markedly reduced compared to that of PrP^C^, suggesting volumetric differences between these two isoforms. Biophysical, structural and molecular dynamics studies have revealed that the transition from the α-helical to the β-sheet enriched conformation had profound effects on recombinant PrP hydration and packing [33], [34], [35], these two properties directly affecting the volume of a protein. Caveolin-1, a major, -supposedly oligomeric [36], [37], [38]- component of ubiquitous plasma membrane invaginations termed caveolae [39] segregated, at the equilibrium, from monomeric lipid raft resident proteins such as Thy1 and flotillin, further supporting the overlooked notion that oligomerization could markedly alter protein density.

The existence of two PrP^Sc^ density peaks is intriguing and will obviously deserve further investigations. First, this may reflect PrP^Sc^ molecular mass variations within the brain, which can affect density [40]. Endogenously, PrP^Sc^ is differentially trimmed by certain nerve cell subpopulations [41], [42], [43]. The resulting amino-terminal deletion may additionally affect PrP hydration and cavity distribution [44]. Besides, PrP^Sc^ aggregation state polymorphism may contribute to differential hydration, as observed with β_2_-microglubulin fibrils [45]. There is no real consensus over the volumetric properties of amyloid fibrils. They can be associated to compaction or less packed structures [46], [47]. PrP^Sc^ binding to ligands, some being known to target the N-terminal part of PrP (for review [48]) could also affect PrP density [33]. The strain-dependent proportions of PrP^Sc^ at the peaks of density would be consistent with these hypotheses: prion strains target specific brain area and can exhibit differential PrP^Sc^ processing [42], [43], different aggregation states [20] and binding to specific ligands might be strain-dependent [49].

Given all the possible reasons for heterogeneous PrP^Sc^ density, the alteration in the PrP^Sc^ density profile of *fast* 127S (**Figure 4C**) and *slow* LA19K strains (**Figure S3**) upon addition of digitonin to the solubilization procedure remains difficult to explain. Its specificity of action as compared to other cholesterol-depleting agents, its absence of effect on the SV properties of PrP^C^ and PrP^Sc^ (**Figure S4**) together with a yield of protein solubilization equal or inferior to that achieved with dodecyl maltosite [20], [50], [51] are strong arguments against an increase in the solubilization yield. Thus differences of densities are more likely to reflect differences in the properties of the bound-detergent species.

At the equilibrium, PrP^Sc^ and infectivity sedimented relatively congruently, whatever the prion strain studied, yet infectivity was not distributed in two clearly distinct peaks of densities like PrP^Sc^. There are differences in the infectivity density values previously published [52], [53], [54] and ours, which are likely explained by the use of different starting material, distinct gradient medium and the degree of solubilization achieved. Our density values found for caveolin, -a protein recovered in fractions nearby PrP^Sc^ and infectivity-, are consistent with those published [55]. Importantly, the density distribution of PrP^Sc^ and infectivity from the 127S *fast* strain were jointly altered by digitonin. This result strengthened the truly physical association between PrP^Sc^ and infectivity with respect to the density of the *fast* prion strain assemblies. Collectively, these data indicate that a small size and not a low density accounts for the hydrodynamic behavior of the *fast* strains most infectious component by SV. Keeping in mind all the uncertainties in determining the molecular mass by SV, we estimated previously that these assemblies might correspond to a pentamer of PrP, if constituted of PrP only [20]. However this value might be underestimated as we showed here that PrP density/volumetry has been dramatically altered by its refolding into PrP^Sc^.

There is clear evidence that a variable, strain-dependent proportion of PrP^Sc^ can be fairly sensitive to PK treatment [56], [57], [58], [59]. Such PrP^Sc^ species have been proposed to be formed of low molecular weight aggregates [31], [32]. PK-sensitive PrP^Sc^ has been shown to support a substantial fraction of infectivity [59], [60], -although this might be strain dependent [57], [61]-, and to have a substantial in vitro converting activity [31], [62]. The PrP^Sc^ content associated with *fast* strains such as 127S or LA21K *fast* resists fairly harsh PK treatment conditions, notably compared to Nor98/atypical scrapie ([21] and unpublished data). Subjecting LA21K *fast* crude brain homogenate to a PK treatment destroying 99% of PK-sensitive PrP^Sc^ infectivity [59] prior to SV fractionation negligibly affected the infectivity associated to the small sized assemblies, as measured reproducibly by the incubation time bioassay. These results are consistent with the inability to detect thermolysin-resistant PrP^Sc^ [20], that might be indicative of the presence of PK-sensitive molecules [57], [63]. Counter-intuitively, the infectivity of LA21K *fast* higher size PrP^Sc^ assemblies appeared more sensitive to the PK treatment than that of the smaller ones, suggesting possible differences in the tertiary structure between the 2 populations of assemblies. These data reinforce the view [20] that PK sensitivity does not inversely mirror the size of PrP^Sc^ assemblies, at least for certain prion strains.

Here we observed a strict quantitative correlation between the *fast* prion strains aggregates templating activity, as measured by the conversion of ovine PrP^C^ by PMCA, and their infectivity as measured by mouse incubation time bioassay or replicating activity in cell culture. The templating activity of the smallest size PrP^Sc^ aggregates particles was 2–3 logs over that of the bulk of higher sized PrP^Sc^ aggregates. Whether this is due to their size, -the smaller, the swifter to polymerize [64]-, or to their specific infectivity remains clearly an open, overlooked question [62] we are currently addressing. Given the superior templating activity of the smallest size PrP^Sc^ aggregates, further studies are ongoing to examine whether the SV profile of PMCA-generated PrP^Sc^ would be enriched in such assemblies and thus would differ from that of the original brain material. This would be consistent with recent observations suggesting a preferential selection of certain PrP^Sc^ conformers during PMCA reactions [65].

The longest PrP^Sc^ polymers (assuming they are linear) could conceivably [66], [67], [68] generate numerous converting pieces as active as the small size oligomers, provided they can be fragmented by the sonication and the beads used in PMCA [69]. They also exhibit low conformational stability values (**Table S1**), as assayed by denaturation assay [70], a characteristic believed to increase the rate of polymer fragmentation [71], [72]. As the main aggregate type in the *fast* strains, they were expected to exhibit the best converting activity. Having actually found the opposite situation raises the intriguing possibility that the most infectious and the most aggregated PrP^Sc^ populations identified by SV might not derive from the same polymerization pathway, as observed with recombinant PrP oligomers [73] and other protein oligomers [74], [75] or, alternatively, that an increase in the polymer size led to an irreversible loss of converting activity. It also suggests that the proposed pivotal role of fibril breakage [6], [7], [72] in hastening fibril growth is a specific property of certain macromolecular assemblies, at least for prion.

The low PMCA activity of the largest PrP^Sc^ assemblies further add to the discrepant impact of the overall stability and/or length of PrP^Sc^ aggregates on its conversion potency [25], [42], [62], [76]. A clear and confounding limitation in such studies is that the properties of the biochemically dominant PrP^Sc^ component are taken as the properties of the whole PrP^Sc^ species while it is obvious here that the specific infectivity and templating activity of PrP^Sc^ assemblies can be heterogeneous. Another layer of intricacy would be provided by the strain to strain variations.

Cumulatively (this study and [20]), the specific infectivity and converting activity (the levels of infectivity and of PMCA activity divided by the PrP content) of the *fast* prions PrP^Sc^ aggregates appears essentially supported by a minor fraction (<10%) of PK-resistant oligomers of ≤5 PrP molecules, - a size consistent with that deduced from prion radiation inactivation studies [77], [78] -, whereas the bulk of PrP^res^ (>90%), constituted essentially of 12–30 molecules of PrP [20], showed over 1000-fold lowered activities. A considerable proportion of PrP^res^ generated during the course of the disease might thus have a negligible contribution to prion replication dynamics. The reported converting activities of small-sized, PK-sensitive particles [31], [62] or small size PrP^res^ aggregates fractionated by other methods [79], [80] appeared comparatively low. Although the latter studies were based on *fast* hamster strains, we found that their most infectious particles were also associated with small sized particles, as in the *fast* ovine strains [20]. It is worth mentioning their infectious starting material was composed of artificially aggregated PrP^res^ particles that were sedimented before subsequent disaggregation and fractionation [79], [80]. Such a procedure may have destroyed or permanently altered discrete subpopulations of infectious particles [60], [81].

Together, our findings suggest that prion infectious particle size is strain-encoded and participates in the strain biological phenotype, in particular the incubation period of disease. For the *fast* strains, our findings support discrete oligomers as the most effective template in the proteopathic cascade leading to animal death. Their strong converting properties could provide a quick regeneration of templates to sustain prion replication. Their small size could also favor dissemination and initiation of conversion at distance. Whether the oligomeric forms identified in our study demonstrate a more acute neurotoxicity than the larger size aggregates remains to be determined and is currently assessed using prion permissive primary cultures of neurons [82]. As the most potent inducers of the pathogenesis, these oligomers could be *in fine* the most neurotoxic, incidentally concurring with the view that the oligomers generated during neurodegenerative diseases linked to protein misfolding and aggregation are generally more potent than larger multimers in impairing neuronal metabolism and viability (for reviews [2], [83], [84]).

## Supporting Information

Figure S1 **Statistical comparison of the survival times of mice inoculated with SE fractions.** Statistical analysis (non-parametric Kruskal-Wallis test) was performed using survival times (Table 1) of *tg338* mice inoculated with the indicated fractions (fr; black, upper top fractions; red, peaks of PrP^Sc^ density) from sedimentation at the equilibrium of LA21K *fast* (**A**), LA19K (**B**), sheep BSE (**C**), Nor98 (**D**; 2 experiments) prion strains. *: p<0.05; **p<0.01; ***:p<0.001; ****:p<0.0001; ns: not significant.
(TIF)

Figure S2 **Statistical comparison of Rov-cell based infectious titer of fractions from ovine strains 127S and LA21K** ***fast*** **sedimented at the equilibrium.** Statistical analysis (non-parametric Kruskal-Wallis test) was performed using PrP^Sc^ levels per Rov cell infected with the indicated fractions (fr; black, upper top fractions; red, peaks of PrPSc density) from sedimentation at the equilibrium of LA21K *fast* (**A**) and 127S (**B**). *: p<0.05; **p<0.01; ***:p<0.001; ****:p<0.0001; ns: not significant.
(TIF)

Figure S3 **Immunoblot analysis of PK-resistant PrP^Sc^ from LA19K-infected** ***tg338*** **mouse brain sedimented at the equilibrium, upon additional solubilization with digitonin.** LA19K-infected brain homogenate from *tg338* mice was solubilized in ‘standard’ conditions (black line) or by adding digitonin first (grey line) before SE fractionation. The fractions collected from the gradient were analyzed for PK-resistant PrP^Sc^ content by western blot. The mean levels of PK-resistant PrP^Sc^ per fraction shown are the combined and fit replicates obtained from the immunoblot analysis of n = 2 independent fractionations.
(TIF)

Figure S4 **Sedimentation velocity profile of 127S prions upon additional solubilization with digitonin.** Brain homogenates from *tg338* mice infected with 127S prions were solubilized in the standard conditions (plain line) or by adding digitonin first (dotted line). The material was then fractionated by sedimentation velocity. The collected fractions were analyzed for PrP^C^ (green lines) or PK-resistant PrP^Sc^ (black lines) content by western blot. The levels of proteins shown are the mean of n = 2 independent fractionations.
(TIF)

Table S1 **Guanidine hydrochloride denaturation of PrP^Sc^ associated to ‘fast’ and ‘slow’ ovine prion strains.** Pools of brain homogenates from *tg338* mice infected with ovine prions strains were treated with guanidine hydrochloride (GdnHCl; final concentrations ranging from 0M to 4M) for 1 hour at room temperature. The final concentration of GdnHCl was brought to 0.5 M before samples were digested with PK for 1 hour at 37°C (50 µg/ml final concentration). Samples were methanol precipitated. The pellets were resuspended in Laemmli buffer and denatured at 100°C for 5 min. The amount of PrP^res^ as a function of GdnHCL concentration was determined by digital acquisition of chemiluminescent signals after western blot. It showed a sigmoidal transition. The GdnHCl concentrations found at the half-maximal concentration ([Gdn]_1/2_) were determined from interpolation using a nonlinear least-square-fit. The values presented are the mean ± SEM of n≥4 independent experiments.
(PDF)
