# Supplementary figures and images for: Quaternary Structure of Pathological Prion Protein as a Determining Factor of Strain-Specific Prion Replication Dynamics
Source: PLoS Pathog. 2013 Oct 10;9(10):e1003702. doi: 10.1371/journal.ppat.1003702 (PMC3795044; doi:10.1371/journal.ppat.1003702)

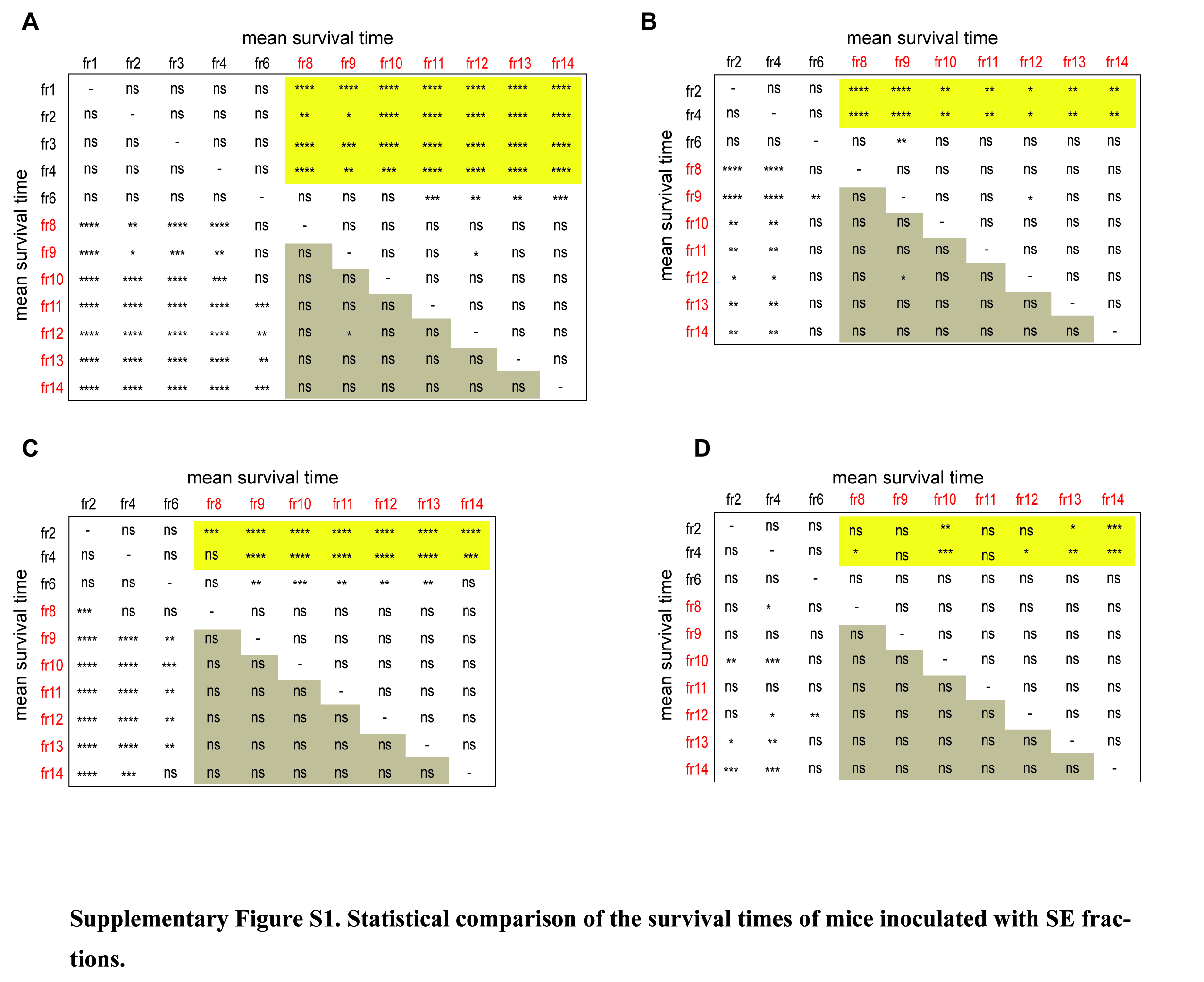

Supplement: Figure S1 — Statistical comparison of the survival times of mice inoculated with SE fractions. Statistical analysis (non-parametric Kruskal-Wallis test) was performed using survival times (Table 1) of tg338 mice inoculated with the indicated fractions (fr; black, upper top fractions; red, peaks of PrPSc density) from sedimentation at the equilibrium of LA21K fast (A), LA19K (B), sheep BSE (C), Nor98 (D; 2 experiments) prion strains. *: p<0.05; **p<0.01; ***:p<0.001; ****:p<0.0001; ns: not significant. (TIF) [file ppat.1003702.s001.tif]

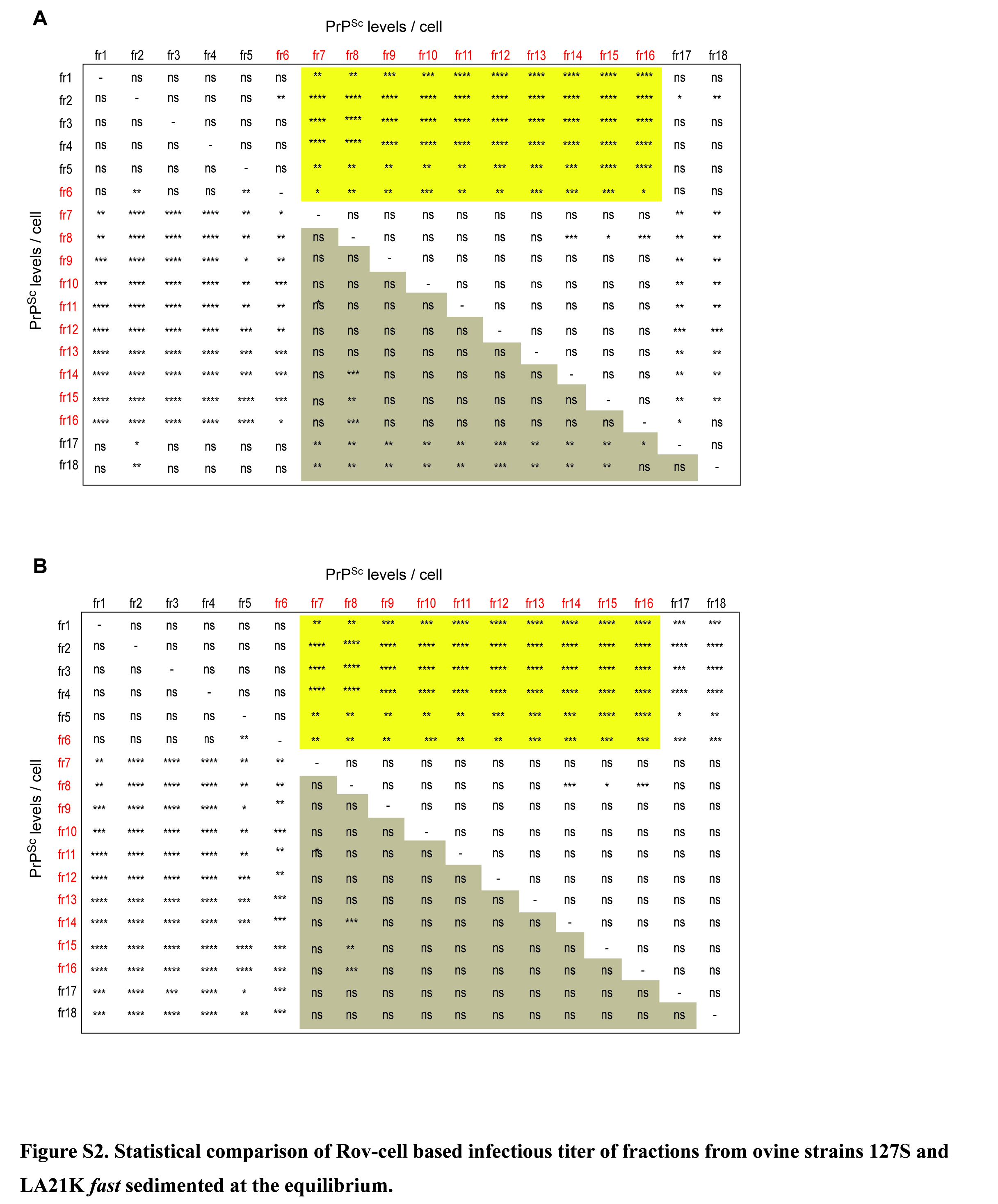

Supplement: Figure S2 — Statistical comparison of Rov-cell based infectious titer of fractions from ovine strains 127S and LA21K fast sedimented at the equilibrium. Statistical analysis (non-parametric Kruskal-Wallis test) was performed using PrPSc levels per Rov cell infected with the indicated fractions (fr; black, upper top fractions; red, peaks of PrPSc density) from sedimentation at the equilibrium of LA21K fast (A) and 127S (B). *: p<0.05; **p<0.01; ***:p<0.001; ****:p<0.0001; ns: not significant. (TIF) [file ppat.1003702.s002.tif]

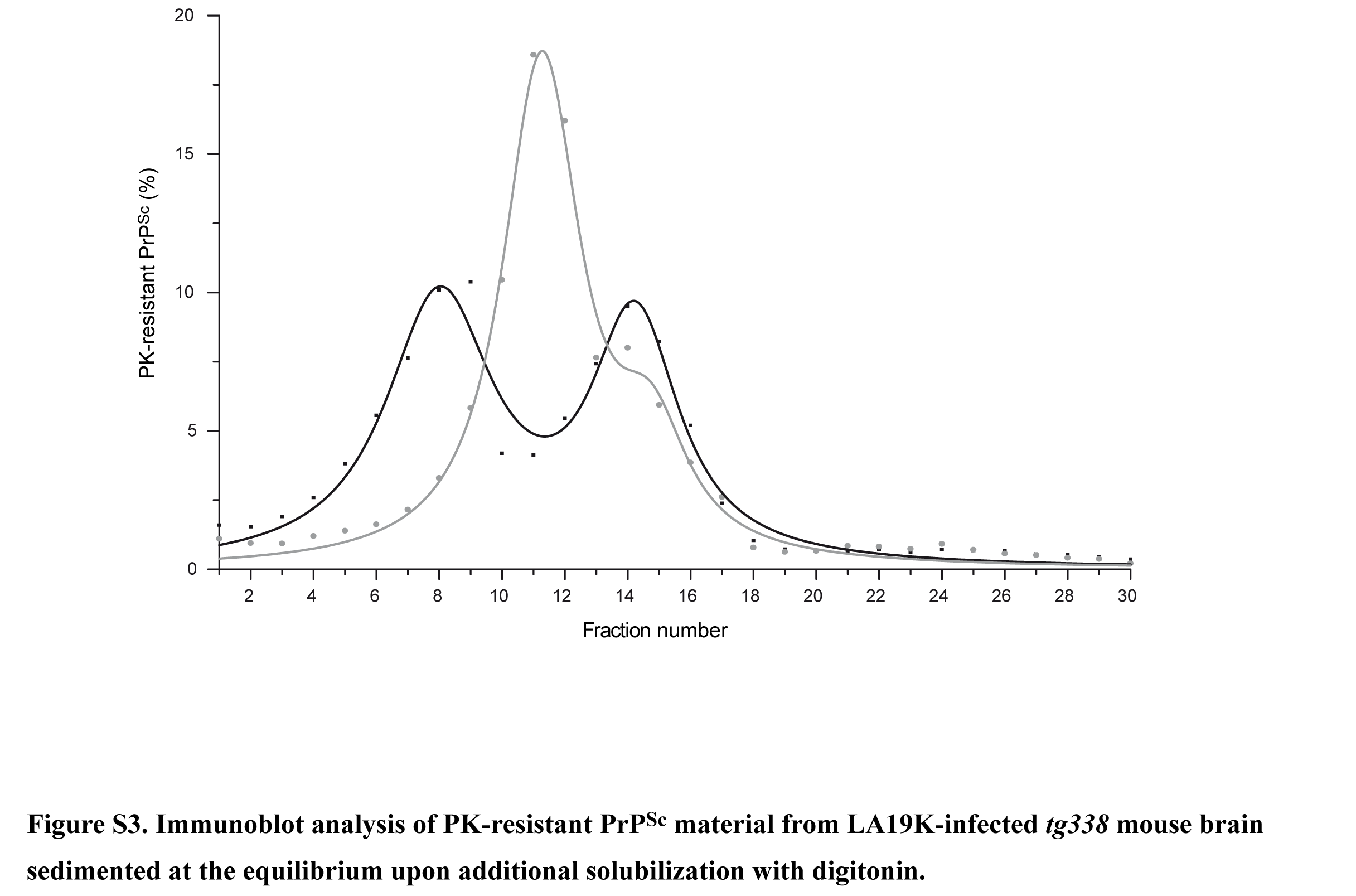

Supplement: Figure S3 — Immunoblot analysis of PK-resistant PrPSc from LA19K-infected tg338 mouse brain sedimented at the equilibrium, upon additional solubilization with digitonin. LA19K-infected brain homogenate from tg338 mice was solubilized in ‘standard’ conditions (black line) or by adding digitonin first (grey line) before SE fractionation. The fractions collected from the gradient were analyzed for PK-resistant PrPSc content by western blot. The mean levels of PK-resistant PrPSc per fraction shown are the combined and fit replicates obtained from the immunoblot analysis of n = 2 independent fractionations. (TIF) [file ppat.1003702.s003.tif]

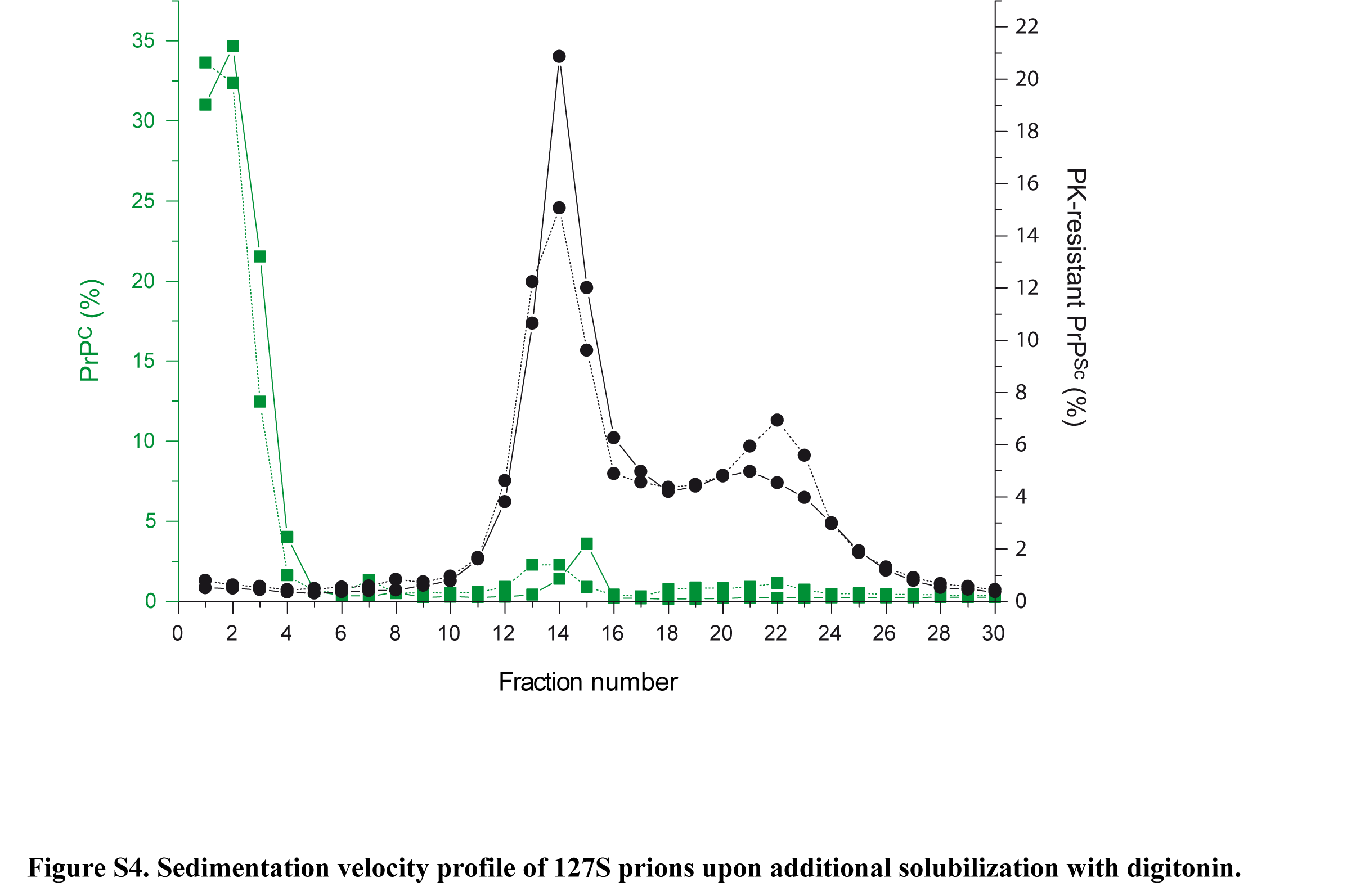

Supplement: Figure S4 — Sedimentation velocity profile of 127S prions upon additional solubilization with digitonin. Brain homogenates from tg338 mice infected with 127S prions were solubilized in the standard conditions (plain line) or by adding digitonin first (dotted line). The material was then fractionated by sedimentation velocity. The collected fractions were analyzed for PrPC (green lines) or PK-resistant PrPSc (black lines) content by western blot. The levels of proteins shown are the mean of n = 2 independent fractionations. (TIF) [file ppat.1003702.s004.tif]
