# Supplementary material for: Quaternary Structure of Pathological Prion Protein as a Determining Factor of Strain-Specific Prion Replication Dynamics
Source: PLoS Pathog. 2013 Oct 10;9(10):e1003702. doi: 10.1371/journal.ppat.1003702 (PMC3795044; doi:10.1371/journal.ppat.1003702)
Supplement: Table S1 — Guanidine hydrochloride denaturation of PrPSc associated to ‘fast’ and ‘slow’ ovine prion strains. Pools of brain homogenates from tg338 mice infected with ovine prions strains were treated with guanidine hydrochloride (GdnHCl; final concentrations ranging from 0M to 4M) for 1 hour at room temperature. The final concentration of GdnHCl was brought to 0.5 M before samples were digested with PK for 1 hour at 37°C (50 µg/ml final concentration). Samples were methanol precipitated. The pellets were resuspended in Laemmli buffer and denatured at 100°C for 5 min. The amount of PrPres as a function of GdnHCL concentration was determined by digital acquisition of chemiluminescent signals after western blot. It showed a sigmoidal transition. The GdnHCl concentrations found at the half-maximal concentration ([Gdn]1/2) were determined from interpolation using a nonlinear least-square-fit. The values presented are the mean ± SEM of n≥4 independent experiments. (PDF) [file ppat.1003702.s005.pdf]

Table S1. Guanidine hydrochloride denaturation of PrP^{Sc} associated to *fast* and *slow* ovine prion strains.

Strain	$[Gdn]_{1/2}$ (mol/l ± SEM)
127S	1.43 ± 0.05
LA21K *fast*	1.64 ± 0.05
LA 19K	2.73 ± 0.11
sheep BSE	2.74 ± 0.04
Nor98	1.86 ± 0.08
